# Screening of differentially expressed genes in the growth plate of broiler chickens with Tibial Dyschondroplasia by microarray analysis

**DOI:** 10.1186/1471-2164-14-276

**Published:** 2013-04-23

**Authors:** Wen-xia Tian, Jia-kui Li, Ping Qin, Rui Wang, Guan-bao Ning, Jian-gang Qiao, Hong-quan Li, Ding-ren Bi, Si-yi Pan, Ding-zong Guo

**Affiliations:** 1 College of Veterinary Medicine, Huazhong Agricultural University, Wuhan 430070, China; 2 College of Veterinary Medicine, Shanxi Agricultural University, Taigu 030801, China; 3 College of Food Science and Technology, Huazhong Agricultural University, Wuhan 430070, China; 4 State Key Laboratory of Agricultural Microbiology, College of Veterinary Medicine, Huazhong Agricultural University, Wuhan 430070, China

## Abstract

**Background:**

Tibial dyschondroplasia (TD) is a common skeletal disorder in broiler chickens. It is characterized by the presence of a non-vascularized and unmineralized cartilage in the growth plate. Previous studies have investigated differential expression of genes related to cartilage development during latter stages of TD. The aim of our study was to identify differentially expressed genes (DEGs) in the growth plate of broiler chickens, which were associated with early stage TD. We induced TD using tetramethylthiuram disulfide (thiram) for 1, 2, and 6 days and determined DEGs with chicken Affymetrix GeneChip assays. The identified DEGs were verified by quantitative polymerase chain reaction (qPCR) assays.

**Results:**

We identified 1630 DEGs, with 82, 1385, and 429 exhibiting at least 2.0-fold changes (*P* < 0.05) at days 1, 2, and 6, respectively. These DEGs participate in a variety of biological processes, including cytokine production, oxidation reduction, and cell surface receptor linked signal transduction on day 1; lipid biosynthesis, regulation of growth, cell cycle, positive and negative gene regulation, transcription and transcription regulation, and anti-apoptosis on day 2; and regulation of cell proliferation, transcription, dephosphorylation, catabolism, proteolysis, and immune responses on day 6. The identified DEGs were associated with the following pathways: neuroactive ligand-receptor interaction on day 1; synthesis and degradation of ketone bodies, terpenoid backbone biosynthesis, ether lipid metabolism, JAK-STAT, GnRH signaling pathway, ubiquitin mediated proteolysis, TGF-β signaling, focal adhesion, and Wnt signaling on day 2; and arachidonic acid metabolism, mitogen-activated protein kinase (MAPK) signaling, JAK-STAT, insulin signaling, and glycolysis on day 6. We validated seven DEGs by qPCR.

**Conclusions:**

Our findings demonstrate previously unrecognized changes in gene transcription associated with early stage TD. The DEGs we identified by microarray analysis will be used in future studies to clarify the molecular pathogenic mechanisms of TD. From these findings, potential pathways involved in early stage TD warrant further investigation.

## Background

**Figure 1:**
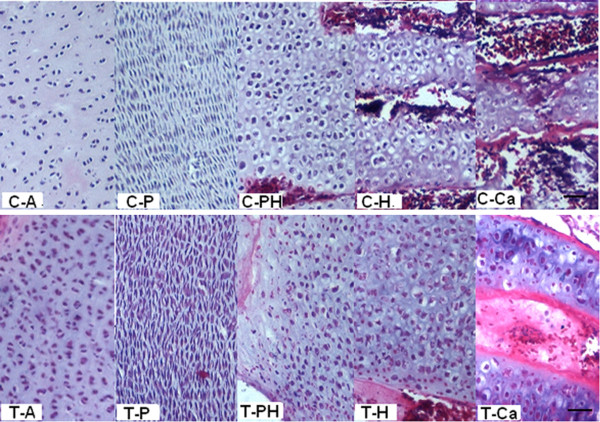
**Histologic characteristics of tibia growth plate development in broiler chickens at day 4 during the experimental period.** C, control; T, thiram-fed; A, articulus zone; P, proliferative zone; PH, pre-hypertrophic zone; H, hypertrophic zone; Ca, calcification zone. Thiram promoted chondrocyte proliferation, resulting in necrosis of a large number of cells, and compressed blood vessels that led to necrosis of blood vessels. The scale bar indicates 200 μm.

Tibial dyschondroplasia (TD) is a common skeletal disorder in broiler chickens characterized by the presence of non-vascularized and unmineralized cartilage in the growth plate. Tetramethylthiuram disulfide (thiram) exerts its cytotoxic effects through membrane damage, mitochondrial injury, inhibition of glutathione metabolism, cell death, and inhibition of angiogenesis. Rath et al. [1] showed that thiram-induced TD is not produced through an increase in chondrocyte multiplication in the transition zone, nor by altering the expression of genes causing the arrest of chondrocytes in a pre-hypertrophic state. It acts by creating metabolic dysfunction that leads to the destruction of blood capillaries in the transition zone chondrocytes. Our previous studies showed that thiram could promote chondrocyte proliferation in the growth plate of chickens, and disturbs the regulation of endochondral calcification and development of normal cartilage. This results in prehypertrophic cell accumulation, angionecrosis, abnormal extracellular matrix synthesis, deferred endochondral calcification, and bone resorption (Figure 1) [2].

Previous studies have investigated the differential expression of genes related to cartilage development during late stage TD using various techniques, such as immunohistochemistry, western blotting, *in situ* hybridization, semi-quantitative polymerase chain reaction (PCR), real-time quantitative PCR (qPCR), and proteomic analyses. These studies revealed abnormal expression of a variety of factors during TD, including extracellular matrix molecules (collagen, osteopontin, osteonectin), growth factors, and hormones [vascular endothelial growth factor (VEGF) and its receptor, hypoxia inducible factor-1 (HIF-1)]. Additionally, matrix metalloproteinases (MMPs), carbonic anhydrase II (CA2), and heat shock proteins (HSPs) were also shown to be affected [3-11]. Recently we generated cDNA libraries for 96-h thiram-induced broiler chicken TD using a suppression subtractive hybridization (SSH) technique, and identified 10 differentially expressed genes (DEGs). Five of these DEGs were found to be upregulated in chicken TD: matrilin-3 (*matn3*); chondromodulin-I (*chm-1*); NADH dehydrogenase (*nadhdh*); cytochrome C oxidase subunit III (*coxIII*); and enolase 1 (*eno1*). Differential expression of these genes may affect cartilage matrix cross-linking, angiogenesis, energy metabolism, and growth regulation [12]. The average cycle of chondrocyte proliferation to terminal differentiation is approximately 21 h; with thiram-induced cytotoxicity accompanied by rapid and significant oxidation, lipid peroxidation, and cell death. Thus, it is likely that the changes that occur during the early stages of TD initiate alterations that are found during late stage TD.

The use of DNA microarray techniques has made it possible for large-scale analysis of gene function and regulation to be conducted. DNA microarrays have been used to study skeletal development in humans and rodents [13-15]. In this study, we screened temporal changes in DEGs during early stages of TD in broiler chickens using an Affymetrix GeneChip, and verified expression patterns at different phases by qPCR. Genetic analyses were used to complement morphological changes and better understand the pathogenic mechanisms of TD.

## Results

### Pathological features of thiram-induced TD

**Figure 2:**
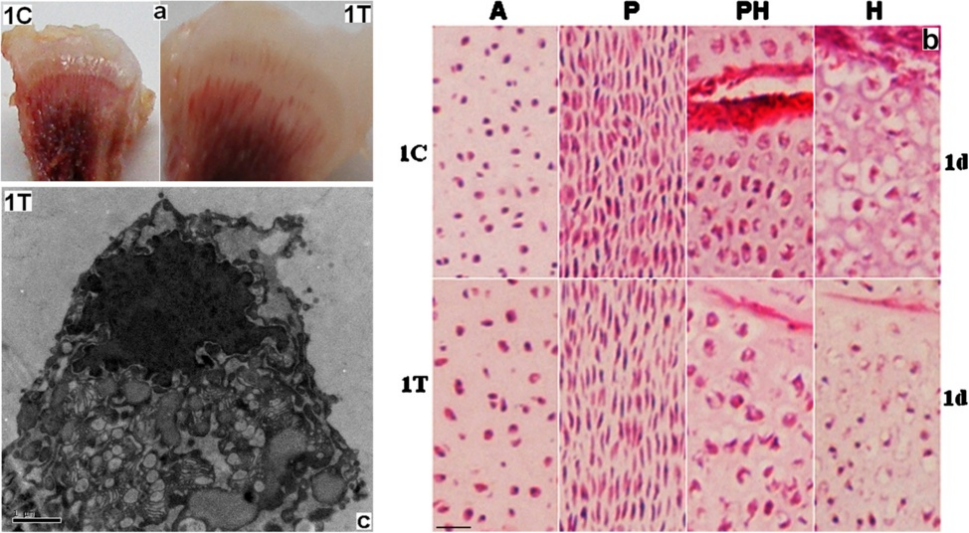
**Pathological characteristics of tibia growth plates due to thiram at day 1.** C, control group; T, thiram-fed group. **a.** Compared with the control group, the growth plate of thiram-treated chickens was thicker. **b.** Histological characteristics of the growth plate of chickens fed thiram. The scale bar indicates 200 μm. A, Articulus zone; P, proliferative zone; PH, pre-hypertrophic zone; H, hypertrophic zone. Chondrocytes at the thickened proliferative and prehypertrophic zones were sparse. Pre-hypertrophic and hypertrophic chondrocytes exhibited pyknosis. The number of empty cartilage capsules was increased, while the number of blood vessels was reduced, with angionecrosis seen at the pre-hypertrophic zone. **c.** Possible apoptotic cells are shown with pyknotic nuclei, enlarged dilated cisternae of the ER that formed a continuous network, and concentrated cytoplasm. The scale bar indicates 1 μm.

Pathologic characteristics of broiler chicken tibia growth plate induced by thiram at day 1 were ascertained (Figure 2). Macroscopic examination revealed that the growth plates of chickens administered thiram were thickened compared with the control group (Figure 2a). Histological characteristics of the growth plates of these chickens were observed by microscopy, and revealed that chondrocytes at the thickened proliferative and pre-hypertrophic zones were sparse. Pre-hypertrophic and hypertrophic chondrocytes exhibited pyknosis. Additionally, the number of empty cartilage capsules was increased and the number of blood vessels decreased, with angionecrosis observed at the pre-hypertrophic zone (Figure 2b). Transmission electron microscopy was used to examine apoptotic cells that contained pyknotic nuclei. These cells exhibited dilated cisternae of the endoplasmic reticulum (ER) that were enlarged and formed a continuous network, with a concentrated cytoplasm (Figure 2c).

### Isolation and assessment of total RNA

Total RNA was isolated from the growth plates of control and thiram-treated chickens on days 1, 2 or 6 post-feeding. Total RNA quality was determined sufficient for subsequent analyses when RNA integrity was 2 (distinct bands corresponding 28S and 18S rRNA at a ratio of 2:1), total RNA concentration was between 2–4 μg/μL, the A_260/280_ ratio was around 2.1, and the A_260/230_ ratio of a sample was near 2.2.

### DEGs at different stages of TD

**Figure 3:**
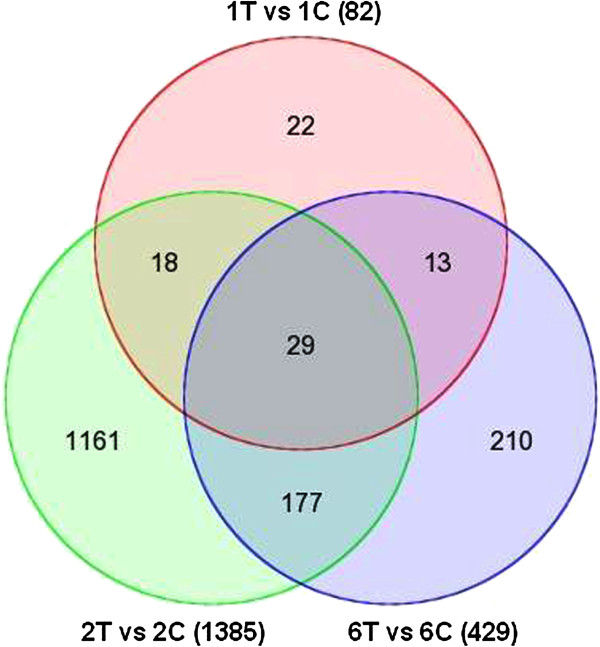
**Analysis of the 1630 transcripts identified on days 1, 2 and 6 using Venn diagrams.** 1C, 2C, and 6C indicates days 1, 2 and 6 for the control group, respectively, while 1T, 2T, and 6T denotes days 1, 2, and 6 for the thiram-fed group, respectively. There were 82, 1385, and 429 DEGs identified on days 1, 2, and 6, respectively. Only 29 DEGs were observed at all three time points. Thirteen DEGs were observed on both days 1 and 6; 18 DEGs were observed on both days 1 and 2; 177 DEGs were observed on both days 2 and 6.

Our one-way ANOVA revealed 1630 DEGs with a minimum 2.0-fold change (*p* < 0.05) in expression level. There were 82, 1385, and 429 DEGs identified on days 1, 2, and 6, respectively (Additional file 1). On day 1, 61 DEGs were upregulated and the remaining 21 downregulated. On day 2, 641 DEGs were upregulated and 744 downregulated, while on day 6, 324 DEGs were upregulated and 105 downregulated. Only 29 DEGs were commonly observed at all three time points; 18 DEGs were observed for both days 1 and 2; 13 DEGs were observed on days 1 and 6; and 177 DEGs were commonly observed for days 2 and 6 (Figure 3). And there were 39, 1086, and 279 DEGs (2.0-fold change, *p* < 0.05) identified on days 1, 2, and 6 by two-way ANOVA (Additional file 2).

### Reliability of microarray screening

Microarray screening assays revealed 18 hybridization maps. All maps showed a regular dot array with excellent signal saturation and homogeneous background. Quality control reports also indicated a stable background around 30, and a noise level of 1.14%. At the experimental setting (α1 = 0.04, α2 = 0.06, τ = 0.015), the marginal signal intensity (M) was approximately 2.2%, thereby confirming the reliability of the microarrays.

To further validate the microarrays, seven up-regulated genes (1.3–73.5-fold) were selected for qPCR analysis. Of these genes, heat shock protein 25 (*hsp25*), and lysyl oxidase (*lox*) expression were significantly upregulated (*p* < 0.05) at days 1, 2 and 6. However, kinectin 1 (*ktn1*), inhibitor of DNA binding 1 (*id-1*), secreted frizzled-related protein 4 (*sfrp4*), cadherin 1 (*cdh1*), and enolase 2 (*eno2*) showed significant (*p* < 0.05) differential expression at two time points. Despite consistent trends of differential expression, the qPCR results did not agree with the microarray data with respect to the range in fold-change (FC) range (Table 1).

**Table 1:** Validation of microarray data by qPCR

**Gene**	**Gene description**	**Microarray FC**			**qPCR FC**		
		**d 1**	**d 2**	**d 6**	**d1**	**d2**	**d6**
*lox*	lysyl oxidase	+3.9	+71.8	+9.6	+4.4	+212.9	+13.8
*hsp25*	heat shock protein 25	+2.1	+43.3	+25.1	+3.4	+298.0	+30.5
*id1*	inhibitor of DNA binding 1	+1.6 ^a^	+2.9	+2.3	+1.1 ^a^	+1.8	+1.2 ^a^
*ktn1*	kinectin 1	+1.5 ^a^	+4.7	+3.6	+1.9 ^a^	+17.1	+5.7
*sfrp4*	secreted frizzled-related protein 4	+2.8	+73.5	+11.6	+2.0 ^a^	+52.2	+11.2
*cdh1*	cadherin 1	+1.3 ^a^	+31.4	+10.9	+2.4 ^a^	+45.4	+3.1
*eno2*	enolase 2	+2.3	+10.5	+4.0	+5.1	+6.2	+1.1 ^a^

+ Represents upregulated. ^a^ Represents *p* > 0.05; d1, d2, and d6 denote the three time points of analysis.

### Cluster analyses of DEGs

**Figure 4:**
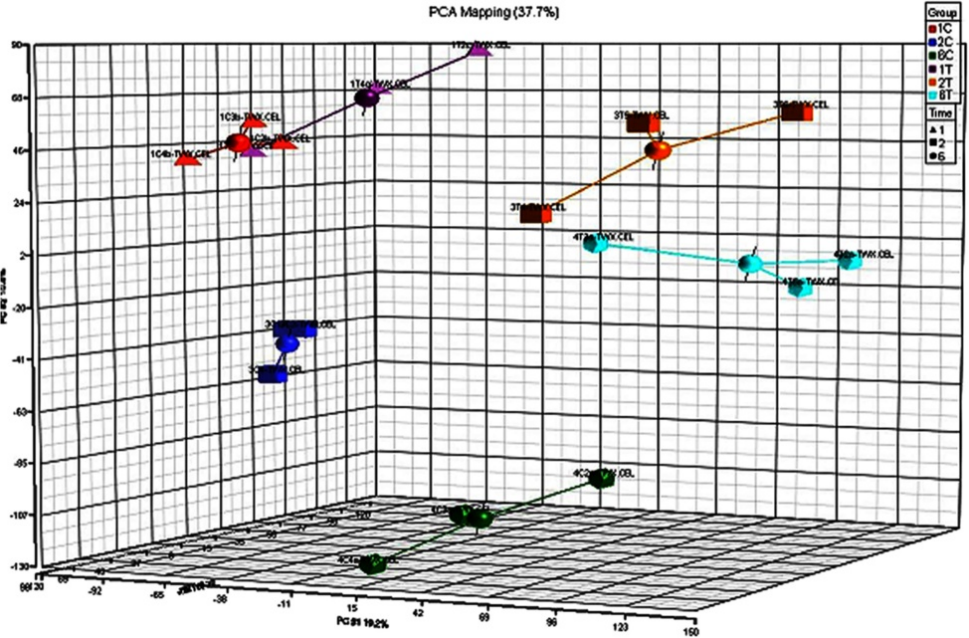
**PCA analysis.** PCA mapping indicated 37.7% similarity at the three time points. The insignificant difference between 1T and 1C indicated that the number of DEGs was small at day 1, with a much greater difference observed at days 2 and 6.

**Figure 5:**
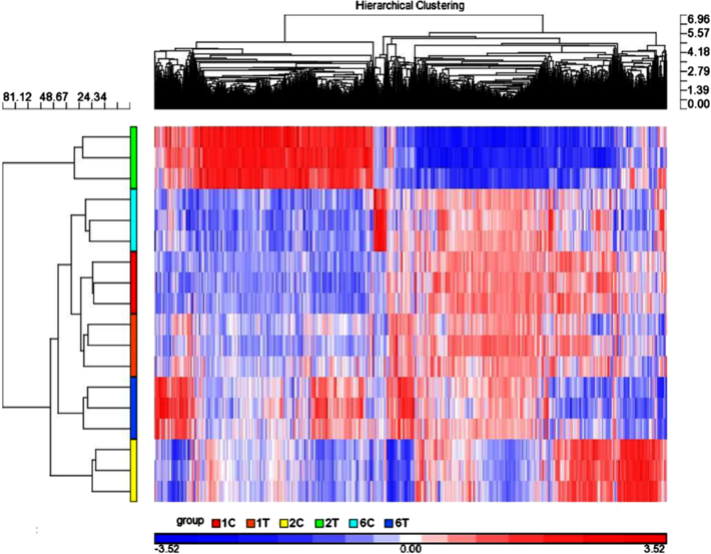
**Hierarchical clustering analysis of differentially expressed genes during TD.** Hierarchical clustering analyses of the 1630 transcripts (*p* < 0.05, FC ≥ 2.0) on days 1, 2, and 6. Red, gray, and blue represent high, average, and absent expression levels of genes, respectively. Each row represents one chicken, and each column refers to a gene. 1C, 2C, and 6C indicates days 1, 2 and 6 for the control group respectively, while 1T, 2T, and 6T denotes days 1, 2, and 6 for the thiram-fed group, respectively.

Principal component analysis (PCA) revealed a similarity of 37.7% at the three time points examined. There was a relatively minor difference in DEGs observed between control and thiram-fed chickens at day 1. However, the differences in DEGs between the two groups were significantly different at days 2 and 6 (Figure 4). A clear expression pattern emerged after hierarchical clustering analyses of the 1630 transcripts (*p* < 0.05, FC ≥ 2.0) on days 1, 2, and 6. Hierarchical cluster analysis also showed that chickens in the control group on days 1, 2, and 6 formed a cluster with similar gene expression patterns. Gene expression patterns of thiram-fed chickens on days 1 and 6 were more similar to those observed in control animals. The gene expression patterns in thiram-fed chickens at day 2 formed a separate cluster with similar gene expression patterns (Figure 5).

### DEGs gene ontology (GO) analyses

**Figure 6:**
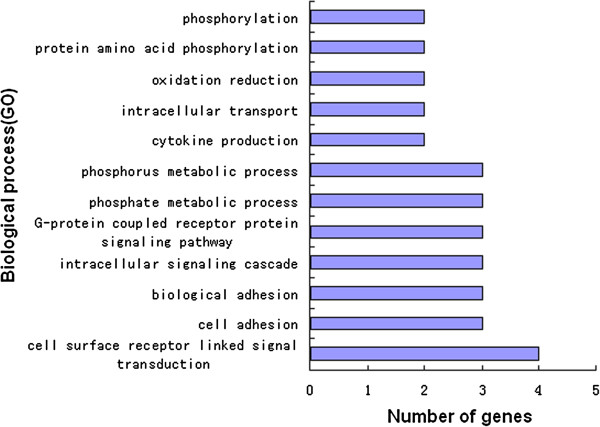
Biological process Gene Ontology (GO) analysis of 82 DEGs on day 1.

**Figure 7:**
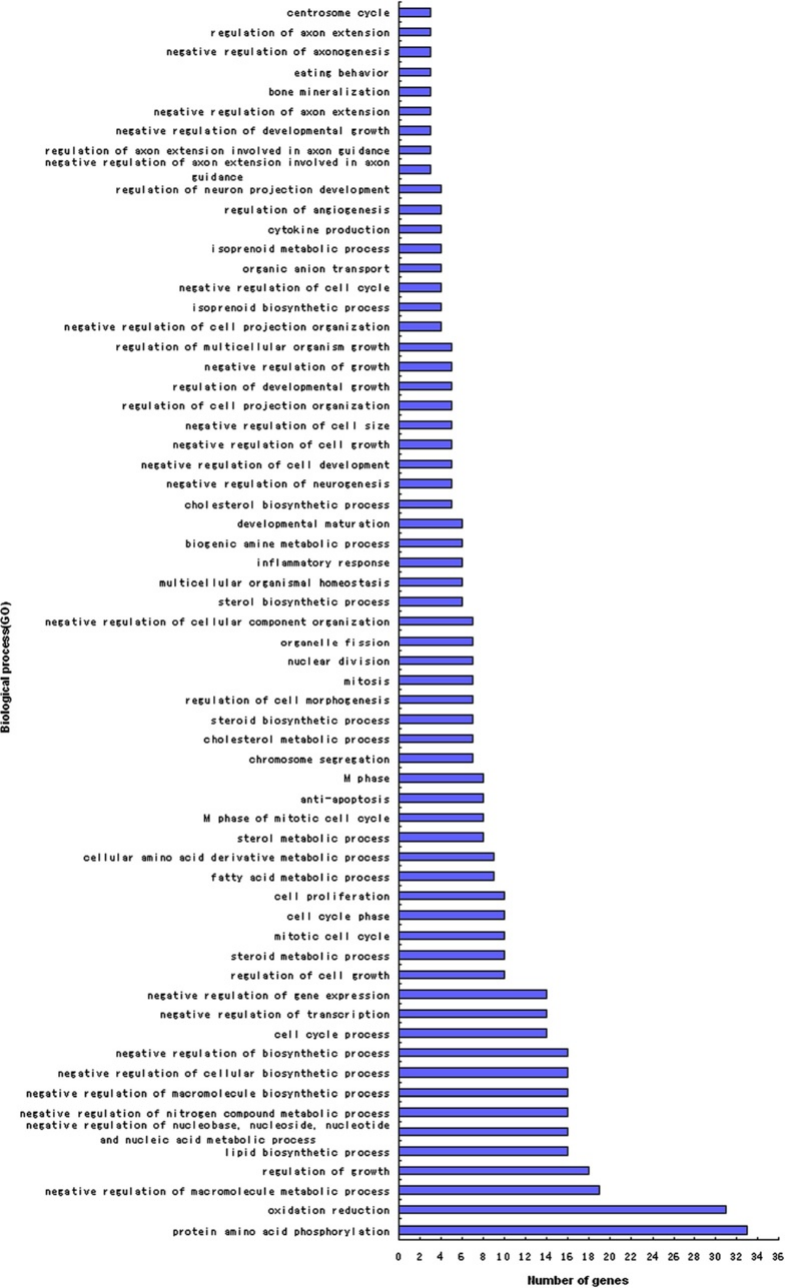
Biological process Gene Ontology (GO) analysis of 1385 DEGs on day 2.

**Figure 8:**
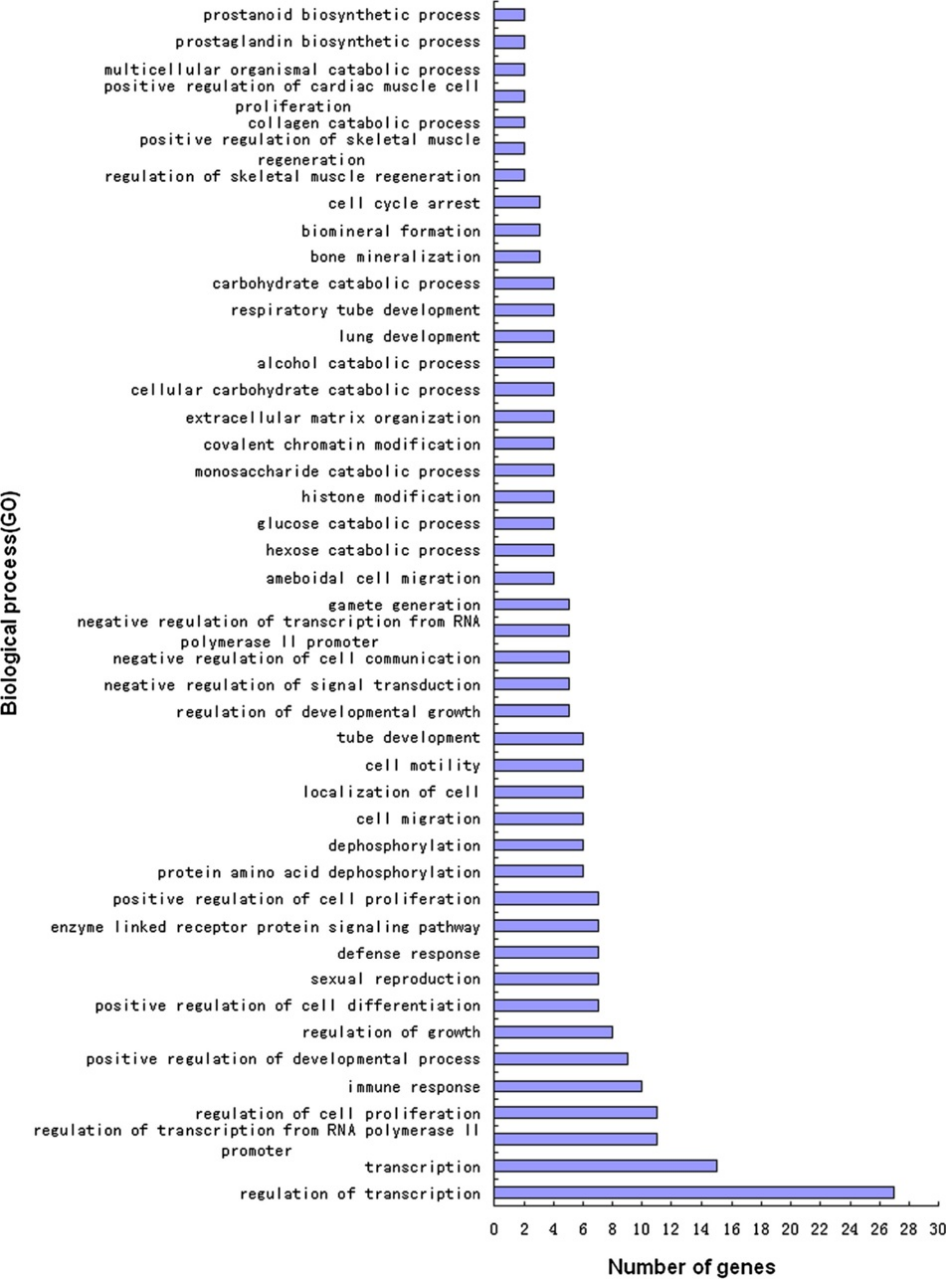
Biological process Gene Ontology (GO) analysis of 429 DEGs on day 6.

Annotation of identified DEGs was carried out using the Database for Annotation, Visualization and Integrated Discovery (DAVID) at the three time points examined. These DEGs were found to participate in a variety of biological processes, such as cytokine production (*p* < 0.05), cell adhesion, intracellular signaling cascades, cell surface receptor linked signal transduction, oxidation reduction and phosphate metabolic processes on day 1 (Figure 6). On day 2 DEGs were associated with transcription regulation, sterol metabolic processes, lipid biosynthetic processes, growth regulation, steroid metabolism, regulation of cell morphogenesis, the mitotic cell cycle, fatty acid metabolism, cellular amino acid derivative metabolism, anti-apoptosis, the cell cycle, positive and negative gene regulation (*p* < 0.05) (Figure 7). On day 6, DEGs were associated with positive regulation of the developmental process, transcription, immune responses, bone mineralization and positive regulation of cell differentiation (*p* < 0.01), proteolysis, protein amino acid dephosphorylation, negative regulation of signal transduction, regulation of cell proliferation, negative regulation of cell communication, and catabolic processes (*p* < 0.05) (Figure 8).

### Potential metabolic pathways related to TD

A number of metabolic pathways were possibly related to TD development between days 1 and 6. Pathways associated with DEGs were: neuroactive ligand-receptor interaction (*p* < 0.05); the phosphatidylinositol signaling system; calcium signaling pathways; and the MAPK signaling pathway on day 1. Synthesis and degradation of ketone bodies, ether lipid metabolism, terpenoid backbone biosynthesis, JAK-STAT, and steroid biosynthesis (*p* < 0.05), GnRH signaling pathway, ubiquitin mediated proteolysis, TGF-β signaling, MAPK signaling, VEGF signaling, focal adhesion, Wnt signaling, and regulation of the actin cytoskeleton were all pathways associated with DEGs identified on day 2. Arachidonic acid metabolism (*p* < 0.01), the MAPK signaling pathway (*p* < 0.05), JAK-STAT, glycolysis, Adipocytokine signaling pathway, insulin signaling, cytokine-cytokine receptor interaction, TGF-β signaling, focal adhesion and regulation of the actin cytoskeleton were associated with DEGs on day 6. Of these DEGs, the expression level of secreted frizzled-related protein 4 (*sfrp4*) was upregulated 2.8-, 73.5-, and 11.6-fold on days 1, 2, and 6, respectively. The expression level of cadherin 1 (*cdh1*) was upregulated 31.4- and 10.9-fold on days 2 and 6, respectively. Similarly, expression of enolase 2 (*eno2*) was upregulated 2.3-, 10.5-, and 4.0-fold on days 1, 2, and 6, respectively.

## Discussion

Previous studies of TD have focused on the late stages; however, in this study we have reported the pathological changes of thiram-induced TD at earlier stages. Our findings show that the growth plate in chickens given thiram was thickened, with a decreased number of chondrocytes at the thickened proliferative and pre-hypertrophic zone. The pre-hypertrophic and hypertrophic chondrocytes exhibited pyknosis. We also observed an increased number of empty cartilage capsules and a decreased number of blood vessels, in addition to angionecrosis at the pre-hypertrophic zone. Transmission electron microscopy analysis of apoptotic cells revealed pyknotic nuclei, and dilated cisternae of the ER that were enlarged and formed a network structure; also, the cytoplasm was concentrated.

Biological process annotation revealed that the DEGs at day 1 following feeding with the thiram diet were associated with cytokine production that primarily involved the neuroactive ligand-receptor interaction pathway. The downregulated genes included chemokine (C-C motif) receptor 6 (*ccr6*), prostaglandin E receptor 4 (subtype EP4) (*ptger4*), arginine vasopressin receptor 2 (*avpr2*), and proprotein convertase subtilisin/kexin type 5 (*pcsk5*), while 5-hydroxytryptamine (serotonin) receptor 7 (*htr7*), and opiate receptor-like 1(*oprl1*) were upregulated.

Genes screened on day 2 revealed the involvement of important biological processes such as sterol metabolism, lipid biosynthesis, steroid metabolism, and terpenoid backbone biosynthesis in particular. Terpenoid, also called isoprenoid, plays an important role during cell growth. Ether lipids, also called plasmalogens or 1-O-alkyl lipids, are ubiquitous and sometimes form major parts of cell membranes in mammals. Differences between the catabolism of ether glycerophospholipids by specific phospholipases might be involved in the generation of lipid second messenger systems, which are important in signal transduction. Ether lipids can also act directly in cell signaling, as the platelet-activating factor is an ether lipid signaling molecule that is involved in leukocyte function in mammalian immune systems. Another possible function of plasmalogen ether lipids are as antioxidants to protect against oxidative stress, therefore these lipids might play a role in serum lipoprotein metabolism. This antioxidant activity comes from the enol ether double bond that is targeted by a variety of reactive oxygen species [16]. Sterols are natural active compounds that form major parts of the cell membranes and are precursors of hormones, vitamin D and sterol compound. Thus, downregulation in the expression of synthases is likely to contribute to the progression of TD.

Analysis of gene expression on day 6 revealed that the biological processes affected were associated with positive regulation of developmental processes involved in arachidonic acid metabolism and the MAPK signaling pathway. Arachidonic acid is the direct precursor of active compounds such as prostaglandin E2, prostaglandin I2 and leukotrienes C4 (LTC4). These play important roles in the regulation of lipoprotein metabolism, cell development, blood vessel elasticity and white blood cell function.

From these GO annotations at all time points, we deduced that the cytotoxic effects of thiram resulted in membrane damage, loss of enzymatic activities, and loss of protein receptor function, especially cysteine-rich proteins. This is likely to influence signal transduction, thereby leading to cell apoptosis and inhibition of pathological angiogenesis.

Our analyses also suggest that thiram-induced TD at the early stages is primarily associated with multiple metabolic pathways, focal adhesion, Jak-STAT, adipocytokine signaling pathway, GnRH signaling pathway, oxidative phosphorylation, glycolysis, Wnt, TGF-β, VEGF, and insulin signaling pathways. Metabolic pathways associated with DEGs at days 2 and 6 were identical. Impaired mitochondrial energy metabolism occurred during the initial stages of TD, with glycolysis subsequently induced at later stages of TD. The focal adhesion and JAK-STAT signaling pathways account for most DEGs, where focal adhesion and JAK-STAT may promote chondrocyte proliferation, development, anti-apoptosis, cell survival, and cell motility through Wnt or MAPK signaling pathways. During early stages of TD, many genes were found to have altered expression, due to the cytotoxic effects of thiram, therefore the proliferation of growth plate chondrocytes and metabolism were altered.

Horvat-Gordon *et al*. [17] studied gene expression in chondrocytes isolated from the proliferative and hypertrophic zones of the avian growth plate using microarrays. They identified a number of genes associated with chondrocyte hypertrophy. Our preliminary analyses of the seven verified DEGs revealed that lysyl oxidase (*lox*), which was upregulated, participates in crosslinking of extracellular collagen *via* oxidative deamination of lysine or hydroxylysine [18,19]. LOX also plays important roles in cell adhesion/proliferation/migration, the intracellular signal response, and malignant processes. Moreover, LOX reenters cells and aggregates around the nuclei after oxidative changes [20]. The LOX propeptide (LOX-PP) inhibits smooth muscle cell signaling and proliferation, thereby providing a feedback mechanism to inhibit pathological angiogenesis [21]. Heat shock protein 25 (Hsp25) acts as a molecular chaperone to stabilize proteins [22-24], maintain membrane integrity, protect nucleic acids, and stabilize the cytoskeleton [25]. Hsp25 is a marker of differentiated odontoblasts [26-29] and also participates in the MAPK signaling pathway [30]. Additionally, the roles of Hsp25 in osteoblast signaling have also been widely studied [31-34]. The ID-1 protein is a member of the ID protein family that has been implicated in the regulation of cellular differentiation, cell cycle progression, senescence, and apoptosis. Moreover, ID-1 also activates VEGF by enhancing the stability and activity of hypoxia-inducible factor-1a [35,36].

Kinectin 1 (KTN1) is a receptor protein in the reticular membrane. KTN1 mainly participates in intracellular vesicle transport and can also regulate protein synthesis in eukaryotic cells by anchoring the elongation factor-1 complex onto the ER [37]. Tamamura *et al*. [38] suggested that Wnt/β-catenin signaling regulates chondrocyte phenotype, maturation, and function during cartilage development. Such regulation is critical for growth plate formation, cartilage boundary definition, and endochondral ossification. Over-expression of SFRP4 may inhibit the proliferation of osteoblasts by antagonizing Wnt signaling [39]. SFRP4 may also cause CDH1 over-expression and thus affect cell adhesion and impair angiogenesis [40]. In addition, hypoxic conditions disrupt metabolism and ultimately results in ENO2 and ENO1 over-expression [12].

## Conclusion

We identified global gene expression changes during early stages of thiram-induced TD. Downregulation of prostaglandin E receptor 4 (subtype EP4) (*ptger4*) and arginine vasopressin receptor 2 (*avpr2*) on day 1 directly influenced signal transduction and blood vessel elasticity. On day 2, downregulation of a variety of synthases altered the production of lipid compounds that are precursors of hormones, vitamin D, PTGD, and PTGE, all of which likely have an important role in angiogenesis, regulation of transcription and cell proliferation, and bone development. Taken together, the findings from this study contribute potential insights into therapies for TD, and also provide fundamental information regarding the pathological mechnisms involved during the early stages of TD.

## Methods

### TD induction and tissue collection

Broiler chickens (7 days old) were randomly divided into two groups. After fasting overnight, animals were fed a regular diet (control group) or a diet containing 100 mg/kg thiram for 48 h (thiram-fed group) to induce TD, as previously described [12]. Eight birds from each group were sacrificed by cervical dislocation under ether anesthesia on days 1, 2, and 6 after commencement of the experiment. The cartilage growth plates from individual birds were harvested, immediately frozen in liquid nitrogen, and stored at −70°C. All procedures were performed according to protocols approved by the Biological Studies Animal Care and Use Committee of Hubei Province, China.

### Gross and microscopic TD lesions

On days 1, 2 and 6, tibiae were observed and recorded, and then immediately removed from two groups of chicks. Samples were fixed overnight in 4% paraformaldehyde or 2.5% glutaraldehyde in phosphate-buffered saline (PBS) at 4°C. Serial histological sections (4-μm thickness) were prepared after samples had been fully decalcified in 10% ethylenediaminetetraacetic acid (EDTA) decalcifying fluid, dehydrated, embedded in paraffin wax, and then stained with hematoxylin and eosin. Sections were examined by light microscopy. For transmission electron microscopy, pre-fixed samples were post-fixed in 2% OsO4, dehydrated, and embedded in epoxy resin. Ultrathin sections were stained with uranyl acetate and lead citrate, and observed with a HITACHI H-7650 transmission electron microscope at 80 kV and a Gatan 832 CCD camera.

### RNA extraction

Individual cartilage growth plates collected from each group of birds were homogenized in TRIzol reagent to extract total RNA according to an improved method. RNA integrity and concentration were evaluated using denaturing formaldehyde gel electrophoresis and a Nanodrop 2000 analyzer (Thermo Scientific), respectively.

### Microarray hybridization and signal processing

Total RNA samples from18 chickens (three individual chickens from each group on days 1, 2 and 6) were sent to GeneTech Biotech for hybridization to chicken Affymetrix GeneChips (Affymetrix). Briefly, total RNA (500 ng) was purified using a QIAGEN miRNeasy kit. The GeneChip IVT Labeling Kit (Affymetrix) was used for synthesis of biotin-labeled cRNA. Labeled cRNA (15 μg) was hybridized to the GeneChip Chicken Genome Array at 45°C for 16 h. GeneChips were washed and stained with a GeneChip Fluidics Station 450 (Affymetrix) using a standard protocol, and probe arrays were scanned using a Scanner 7G. Quality control (QC) data were obtained using Expression Console software. Principal component analysis (PCA) and histogram cluster analysis were conducted with Partek GS 6.4. Identification of DEGs was also conducted using Partek GS 6.4 through by one way ANOVA and two way ANOVA. The *p*-value cutoff for DEGs was set at 0.05. The adjusted p-value was computed by the false discovery rate (FDR) using Partek GS 6.4, and FDR of approximately 5% was set as a threshold. DEGs were subjected to hierarchical clustering using Cluster (version 3.0) and visualized with TreeView (version 1.6; Stanford University). The identified DEGs were analyzed for GO and biological pathways using DAVID (http://david.abcc.ncifcrf.gov).

### Verification of identified DEGs by qPCR

Total RNA samples used for qPCR verification had also been used in the microarray analyses. Transcripts from each sample were amplified in triplicate and detected using a SYBR Green PCR Master Mix (Applied Biosystems). All primers used (Table 2) were synthesized by Genery Biotechnology. The *RPS16* gene was used as the internal control for normalization, while the reference gene *β-actin* was used to verify the differences in expression levels. Data from qPCR assays were analyzed with Sequence Detector software (version 1.3.1; Applied Biosystems) and were performed by a variance analysis (ANOVA). A *P*-value less than 0.05 was considered significant.

**Table 2:** Primers used for qPCR analysis

**Gene**	**Gene descriptions**	**Primers sequence (5′-3′)**	**Target size (bp)**	**Tm (°C)**
*lox*	lysyl oxidase	Forward: TACGTGCAGAGGATGTCCATGT	114	60
		Reverse: TCTCAGGAGCACTCGGTTGTC		
*hsp25*	heat shock protein 25	Forward: AGGAGTGTGCCCATCCAGGT	109	60
		Reverse: GATGCAGACCGTTGTTCCGT		
*id1*	inhibitor of DNA binding 1	Forward: GGGGTCATTGCCGACATT A	115	60
		Reverse: AGGGGGGACTCAGGAATGTA		
*ktn1*	kinectin 1	Forward: CGGTGAATCTTAACCAGGATGTAG	134	60
		Reverse: TGCTATCCTCAGATCAGCGATT		
*sfrp4*	secreted frizzled-related protein 4	Forward: GCTGAATCTATCTGCTGTTGGG	136	60
		Reverse: CTAACACTGTAAGCATATTTCTGGC		
*cdh1*	cadherin 1	Forward:TGAGAAGCAGATACTGAGCATTGTG	122	60
		Reverse: GCTCATCTTGGCCCCTTATCTC		
*eno2*	enolase 2	Forward: GTGTGCGTGTGTGTAGGTGTATGT	113	60
		Reverse: AGTGCTCAGAACGGAAGGAAGA		
*rps16*	ribosomal protein S16	Forward: ACAAACTGCTTGAACCTGTCCTC	127	60
		Reverse: GCTTTGGAAATAGCTTGACGG		

## Competing interests

The authors declare that they have no competing interests.

## Authors’ contributions

WX carried out the molecular genetic studies, sequence alignment, designed the study, performed the statistical analyses, and drafted the manuscript. P, R and JG extracted the total RNA. DR, SY, and DZ conceived the study, participated in its design and coordination, and helped draft the manuscript. JK, GB and HQ provided helpful discussion and advice. All authors read and approved the final manuscript.

## Supplementary Material

Additional file 1 **We identified 1630 transcripts as being differentially expressed in the growth plate on days 1, 2 and 6 with one way ANOVA.** The 1C, 2C, and 6C designations refer to the control group on days 1, 2, and 6, respectively. The 1T, 2T, and 6T designations refer to the thiram-fed group on days 1, 2 and 6, respectively. We have listed 82 transcripts in sheet 1T *vs.* 1C, 1385 transcripts in sheet 2T *vs.* 2C, and 429 transcripts in sheet 6T *vs.* 6C. A FC (gene expression level following administration of the thiram-containing diet compared with the control) greater than 2.0 represents upregulation, while an FC less than or equal to 2.0 represents downregulation. Ajusted *p* value was computed by the false discovery rate (FDR) of approximately 5% using Partek GS 6.4. Click here for file

Additional file 2 **We identified 1222 transcripts as being differentially expressed in the growth plate on days 1, 2 and 6 with two way ANOVA.** The 1C, 2C, and 6C designations refer to the control group on days 1, 2, and 6, respectively. The 1T, 2T, and 6T designations refer to the thiram-fed group on days 1, 2 and 6, respectively. We have listed 39 transcripts in sheet 1T *vs.* 1C, 1086 transcripts in sheet 2T *vs.* 2C, and 279 transcripts in sheet 6T *vs.* 6C. A FC (gene expression level following administration of the thiram-containing diet compared with the control) greater than 2.0 represents upregulation, while an FC less than or equal to 2.0 represents downregulation. Ajusted *p* value was computed by the false discovery rate (FDR) of approximately 5% using Partek GS 6.4. Click here for file
